# Expression of Two Key Enzymes of Artemisinin Biosynthesis FPS and ADS genes in *Saccharomyces cerevisiae*

**DOI:** 10.34172/apb.2021.019

**Published:** 2020-11-07

**Authors:** Elfahmi, Rizqiya Astri Hapsari, Tamara Chrysanthy, Vaniarta Synthiarini, Fifi Fitriyah Masduki, Agus Setiawan, Toshiya Muranaka

**Affiliations:** ^1^School of Pharmacy, Bandung Institute of Technology, Bandung, Indonesia.; ^2^University Centre of Excelence - Nutraceutical, Bioscience and Biotechnology Research Centre, Bandung Institute of Technology, Bandung, Indonesia.; ^3^Biochemistry Division, Chemistry Department, Faculty of Mathematic and Natural Sciences, Bandung Institute of Technology, Bandung, Indonesia.; ^4^Departement of Biotechnology, Graduate School of Biotechnology, Osaka University, Japan.

**Keywords:** Amorpha-4,11-diene synthase, Artemisinin, Farnesyl phosphate synthase, Malaria, Saccharomyces cerevisiae

## Abstract

***Purpose:*** Artemisinin, a secondary metabolite in Artemisia annua is one of primary choice for the treatment of malaria, it is naturally produced in low concentration from this plant. This study was aimed to clone key enzymes of artemisinin production in order to enhance its production through the semi-synthetically production in * Saccharomyces cerevisiae.*

***Methods:*** Two key enzymes in artemisinin biosynthetic pathway which are farnesyl phosphate synthase (*fps*) and amorpha-4,11-diene synthase (ads) genes were transformed into *S. cerevisiae* using pBEVY vector. Successful transformation was checked by polymerase chain reaction (PCR) method and sequencing analysis

***Results:*** Recombinant plasmids which are pBEVY-GU_*ads* and pBEVY_GL_*fps* were successfully constructed. The optimized *ads* gene was amplified using PCR with a couple of primers that are designed in order to provide the homolog recombination between *ads* gene with the expression plasmid of pBEVY-GU respectively. While the *A. annua* optimized *fps* gene was cloned using classical method. Transformants were grown in selective media Synthetic Defined (SD) without leucine for transformants contain plasmid pBEVY-GL_*fps* and media without uracil for transformants contain plasmid pBEVY-GU_*ads*. Confirmation of colonies was done by PCR with primers to amplify *fps* and *ads*. DNA from yeast was isolated from positive colonies then transformed to *E. coli*. Plasmid from *E. coli* was isolated for restriction analysis and sequencing. Protein expression was induced by cultivating the yeast in the media with 2% galactose.

***Conclusion:*** Based on PCR, restriction and sequencing analysis, it could be concluded that *fps* and *ads* genes were successfully constructed, transformed and expressed in S. cerevisiae.

## Introduction

Malaria is a disease caused by a parasite *Plasmodium* sp which is transmitted through an *Anopheles* mosquito bite. There are five main species of malaria caused by parasites such as *Plasmodium falciparum*, *P. vivax*, *P. ovale*, *P. malariae*, and *P. knowlesi*. The symptoms of malaria infection are fever, tremor, nausea, vomiting, and painful. In acute malaria, the complication caused the organ damage and abnormalities of metabolism and blood circulation of patient. This is shown by cerebral malaria, anemia due to hemolysis, hemoglobinuria, disorder of respiration and blood coagulation, hypotension and kidney desease.^1^ According to World Malaria Report 2017, in year 2016, it is estimated around 216 million malaria cases were found in 91 countries in the world.^2^ In commonwealth countries, 128 million cases of malaria were found in 2017.^3^ The main problem for the attempt to eliminate malaria is multi drugs resistance. Drug resistance is the ability of parasite to survive and do multiplication when treated by the same drugs with higher doses.^4^ *P. falciparum* is a parasite which has been resistant to some antimalarial drugs such as chloroquine, sulphadoxine-pyrimethamine, quinine, piperaquine, and mefloquine.^5-8^ Since 2005, WHO has recommended the use of artemisinin-based combination therapies (ACTs) to treat malaria caused by *P. falciparum*. ACTs used the combination of two or more active compounds which have different mechanisms on malaria treatment.

Artemisinin is a terpenoid compound containing 15 carbon atoms with endoperoxide lactone.^9^ Artemisinin is isolated from *Artemisia annua* plant which belongs to family Asteraceae. Besides *A. annua*, other species have also been reported to produce artemisinin which are *A. apiacea*, and *A. lancea*. The highest artemisinin content is produced by *A. annua* which has around 0.01-0.5% dry weight.^10^ Hybridization of variety in central Africa yielded *A. annua* which contains artemisinin around 0.63-0.7% dry weight; however, it is only 40% of the content could be extracted.^11^ The biosynthesis of artemisinin involves 2 main steps. The first step is the production of isoprenoid unit which is a building block for the group of terpenoid compounds. In this step, the precursors of terpenoid are produced including geranyl pyrophosphate, farnesyl pyrophosphate (FPP), and geranyl-geranyl-pyrophosphate. The enzymes involved in this step exist in most organism. In the beginning, isopentenyl pyrophosphate (IPP) and its isomer dimethyl allyl pyrophosphate (DMAPP) are synthesized via two pathways: mevalonic (MVA) and non-mevalonic or methylerythritol phosphate (MEP) pathways. MVA pathway occurs in cytosol, where acetyl CoA is converted into mevalonic acid by HMG-CoA reductase. MEP pathway is found in plastid, starting from reaction between pyruvic acid and phosphoglyceraldehyde yielded 1-deoxy-D-xylulose-5-phosphate (DXP) which is then reduced into 2-C-metthy-D-eritriol-4-fosfat (MEP).^12-14^ MVA and MEP are converted into IPP and DMAPP respectively. IPP and DMAPP are converted to FPP by farnesyl phosphate synthase (*fps*). In the second steps, the cyclization of isoprene involves the enzymes which vary among species.^15^ The cyclization process involves the conversion of FPP into amorpha-4,11-diene by the enzyme amorpha-4,11-diene-synthase (*ads*). Amorpha-4,11-diene will be followed by other enzymatic conversions into artemisinic alcohol, artemisinic aldehyde, aldehyde dihydroartemisinic and dihydroartemisinic acid which are catalyzed by cytochrome P_450_ (*cyp71AV1*), artemisinic aldehyde reductase (*dbr2*), and aldehyde dehydrogenase (*aldh1*) respectively.^13,14,16^ Dihydroartemisinic acid and artemisinic acid are converted into artemisinin through spontaneous oxidative reaction in several steps.^17,18^

Due to the low content of artemisinin in *A. annua* plant, and the chemical synthesis is rather difficult and not economically prospective, the effort to produce artemisinin and its derivatives through biotechnology technique is continuously performed. One of the techniques is the semisynthesis of artemisinin. In this techniques, two steps could be done. The first step is the genetic engineering of microorganism harboring the genes of artemisinin biosynthesis which produce the artemisinin derivative, then continue with the chemical synthesis. The produced precursor through genetic engineering is artemisinic acid which is then converted into dihydroartemisinic acid using a simple reaction. The microorganism used for the genetic engineering are *E. coli* and *S. cerevisiae*.^19,20^ In this research, two of key enzymes encoded genes in biosynthesis artemisinin have been transformed into *S. cerevisiae* in order to prepare engineered yeast to be continued for production of artemisinin and its derivatives.

## Materials and Methods

The materials used in this research included Luria-Bertani (LB) media with composition of 1% NaCl, 1% tryptone, 0,5% yeast extract, and LB agar plate supplemented with 2% bacto agar, MgCl_2_, CaCl_2_, glycerol, ampicillin, restriction enzymes, Gel/PCR Fragment DNA Extraction Kit (Geneaid), QIAprep Spin Miniprep KIT (250), Mix Q5 Taq Polymerase 2X, Dream Taq Polymerase, Dream Taq 10X buffer, DNA template (GenScript pUC *ads*), yeast extract peptone dextrose (YPD) media and YPD agar plate, synthetic defined (SD) media and agar plate. *Artemisia annua* codon optimized of *fps* and yeast codon optimized of ads genes, pGem-T-easy and pBEVY-GL and pBEVY-GU vectors. Primers used to amplify *fps* and *ads* genes are listed in Table 1.

**Table 1:** Sequences of primers for amplification of *fps* and *ads* genes

**Primers**	**Sequence**
FPS-Aa-for(BamHI)	5’-ATGAGTAGCATCGATCT-3’
FPS-Aa-rev(PstI)	5’-CTACTTTTGCCTCTTGT-3’
ADS_MCSII_ gibsonF	5’-AACGTCAAGGAGAAAAAACCCTGGTACCGAGCTCATGTCCTTGACCGAAGAAAAACC-3’
ADS_MCSII_ gibsonR	5’-GGCATAGGAGATCCGCTTATTTAGAAGTGTCGAATTCTTATCAGTGATGGTGATGGTGATGGATAGACATAGGATAGACCAATAAGGATTTG-3’

### Construction of pGEM-T easy fps and pBEVY-GL_fps plasmid

The *A. annua* optimized *fps* gene was amplified using primer *fps* and ligated to pGEM-T easy. The PCR product was thus purified using PCR DNA fragment clean up. As much as 4 μL purified DNA fragment was ligated to 1 μL of pGEM-T easy together with ligase buffer and ligase. The ligation product was transformed to E. coli TOP10 F’. For analysis, the transformants was checked with PCR colony. The positive colony thus cultivated and isolated its recombinant plasmid, followed by restriction analysis and sequencing. Further, the *fps* gene was sub-cloned to pBEVY-GL (a bidirectional yeast expression vector allowing expression of two genes simultaneously under galactose promotor and leucine auxotroph selection marker) by cutting the recombinant plasmid pGEMT-easy_*fps* and circular pBEVY plasmid using restriction enzyme of BamHI and PstI. One µL of each restriction enzyme was mixed with 2 µL of plasmid, 5 µL of buffer Tango 10X, and DNAse and RNAse free water up to 50 µL. The mixture was incubated overnight at 37^o^C. The restriction reaction was analyzed by agarose electrophoresis, and then the targeted band was purified using Gel/PCR DNA Fragments Extraction Kit. The theoretical size of *fps* gene is1038 bp, while pBEVY-GL plasmid is 6631 bp. Furthermore, purified *fps* gene and pBEVY-GL vector were ligated using T4 DNA ligase with ratio 1:10 at 4^o^C for 18 hours then transformed to *E. coli* TOP10F’ competent cells.

### Escherichia coli competent cells

The competent cell of *E. coli* TOP10F’ was prepared as follows: a glycerol stock of *E. coli* cell was inoculated on LB plate supplemented with tetracycline 15 µg/mL. Plate was incubated at 37^o^C, for 14-16 hours (overnight). A colony of *E. coli* was taken and inoculated in 5 mL of LB media without antibiotic and incubated at 37^o^C while shaking on shaker 150 rpm for 14-16 hours. Thus, 1 mL of pre-culture was inoculated into 200 mL of LB media in flask and OD_600_ was monitored every hour using spectrophotometer UV, and every 15 min when OD_600_ has reached 0.2. The cell was cultivated until OD_600_ reached 0.35-0.4 and it was put on ice for 30 min. *E. coli* culture was harvested by centrifugation at 4000 rpm for 15 min at 4^o^C. Supernatant was discarded, and pellet was re-suspended with cold 20 mL of MgCl_2_ 100 mM and washed again with 40 mL of CaCl_2_ 100 mM and stored on ice for 20 min. The cell was harvested and centrifuged again at 3000 rpm for 15 min at 4°C. Supernatant was discarded, and lastly pellet was re-suspended with CaCl_2_ 85 mM and glycerol 15%. Aliquot of 100 µL cells was taken and put in sterile Eppendorf tubes and stored in -80°C.

### Cloning and E. coli transformation

pBEVY-GL_*fps* plasmid was transformed in *E. coli* competence cell using heat shock technique. The cell was incubated in water bath at 42^o^C for 90 seconds, then stored on ice for 2 min. It was added with liquid media without antibiotic and incubated on shaker at 37°C for 1 hour. The cell was harvested and centrifuged at 3000 rpm for 15 min, supernatant was discarded, and pellet was re-suspended with liquid LB media without antibiotic. The cell was then spread on selection LB media with ampicillin 100 ppm and incubated at 37^o^C for 16 hours. For confirmation, the transformant colonies were collected and used as a template for PCR. The master mix for PCR consists of MyTaqTM Red Mix, forward and reverse primers fpsAa-for-BamHI and fpsAa-rev-PstI, each total volume of PCR tube was 10 µL PCR condition which was started by denaturation at 95^o^C for 5 min, continue with 30 cycles of 95°C for 1 min, annealing temperature 55°C for 1.5 min, amplification at 72°C for 1 min. The PCR results were checked by electrophoresis. To assure the successful transformation, the plasmid was checked by restriction and sequence analysis. For restriction analysis, pBEVY-GL_*fps* plasmid was isolated and cut with restriction enzyme 0.2 µL of KpnI 10 unit, 2 µL of recombinant plasmid, buffer KpnI 10X 2 µL, and DNAse and RNAse sterile water up 20 µL. The mixture was incubated at 37^o^C for 1 hour. For sequence analysis, the 20 µL of plasmid was sent to Macrogen (Macrogen Inc., Korea) using forward primer Gal10Pro and reverse primers pHyblex. The sequencing data was analyzed using SeqTrace and Serial Cloner software.

### Preparation of S. cerevisiae electrocompetent cells

Saccharomyces*cerevisiae* BY4741 electrocompetent cell was prepared initially by streaking out the glycerol stock-containing cell in YPD agar and incubated at 30^o^C for three days. A colony was selected and inoculated in liquid YPD media, incubated on shaker 150 rpm at 30°C for 18 hours. As much as 500 µL pre-culture cells was inoculated in 50 mL of YPD media. OD_660_ was measured every 2 hours using spectrophotometer until reaching OD_660_ of 1-1.7. The cells were centrifuged with 4500 rpm for 5 min at 4^o^C. Supernatant was discarded, pellet was re-suspended with cold ddH2O and vortexed and the process was repeated. The cell was then centrifuged again, re-suspended with LiTE-DTT buffer, vortexed and incubated at room temperature for an hour. Afterward, the washed cell was re-suspended with sorbitol 1 M, vortexed, centrifuged at 4500 rpm for 5 min at 4°C. For transformation, *S. cerevisiae* competent cell was put in the tube, then put in the electroporator, run with 1500 V and pulse time 5 min. To cell suspension, sterile liquid YPD media was added, and moved the mixture in micro tube. Cell was harvested and centrifuged at 5000 rpm for 5 min, then re-suspended with a little amount of YPD media and spread it out to SD selection media without leucine for 5 min, then incubated for three days at 30°C. For transformant selection, some colonies were grown in three types of medium which are solid SD media without uracil, without leucine and YPD agar medium, and then incubated at 30°C for 18 hours.

### Expression of fps in Saccharomyces cerevisiae BY4741

Single colony of pBEVY-GL_*fps* containing*S. cerevisiae* BY4741 was cultured in liquid SD media without leucine supplemented with glucose 2%, incubated on shaker 150 rpm at 30°C for 18 hours. OD_660_ of overnight cultures was determined, and then some of them was moved into liquid SD media without luecine with raffinose 2%, incubated on shaker 150 rpm at 30°C until reaching OD_660_ of 0.7-0.8. The production of protein was done in two condition which are induction using raffinose 2% and galactose 2%, incubated on shaker at 30^o^C for 18 hours. For negative control, *S. cerevisiae* wild type in YPD medium was used with the same condition. Expression analysis was done as follows: small amount of cultures were harvested, centrifuged at 5000 rpm for 5 min, re-suspended in NaOH 0,1 M. Suspension was incubated at room temperature for 5 min and centrifuged at 5000 rpm for 5 min. The samples were analyzed using SDS-PAGE acrylamide Theoretically, the size of *fps* is 39,5 kDa.

### Preparation of pBEVY-GU_ads plasmid

Glycerol stocks of *E. coli* which contains pBEVY-GU was inoculated in solid LB media supplemented by ampicillin 100 µg/m, incubated at 37°C for 15-16 hours. Plasmid containing *E. coli* was inoculated in liquid LB media supplemented with ampicillin and incubated on shaker 150 rpm at 37**°**C for 15-16 hours. Plasmids were then isolated using QIAprep Spin Miniprep KIT (250). Isolated plasmid pBEVY-GU was cut with SacI and KpnI and incubated at 37 °C for 15-16 hours. The linearized vector was loaded for electrophoresis, and the band corresponding to the linear plasmid was isolated and purified from the gels using ATP Gel/PCR Fragment DNA Extraction Kit. ADS gen was prepared by PCR using GenScript PUC_ads template, forward primer ADS_MCSII_gibsonF, primer Reverse ADS_MCSII_gibsonR, mix Q5 DNA polymerase 2X, and ddH2O.

### Transformation of pBEVY-GU_ads plasmid into S. cerevisiae

*Saccharomyces cerevisiae* was inoculated in 5 mL of liquid YPD, incubated on shaker 150 rpm at 30^o^C for 15-16 hours, then transferred into 25 mL of media, incubated with the same condition until reaching 0.6 of OD_600_. The cell was harvested and centrifuged at 5000 rpm for 5 min. Cell was re-suspended with water for washing twice. The cell was then re-suspended with 2 mL of cold sorbitol 1M and centrifuged at 5000 rpm for 5 min, and supernatant was discarded. The cell was re-suspended with 2 mL of LiTE and 25 mM DTT, then centrifuged at 5000 rpm for 5 min. supernatant was discarded, furthermore the cell was re-suspended in 2 mL of cold sorbitol 1M, incubated on shaker 5000 rpm for 5 min, supernatant was discarded. The cell was re-suspended in 250 µL of cold sorbitol 1M, and then the suspensions were moved into sterile micro tube each 50 µL. The mixture of plasmid (1 µL) and DNA (4 µL) was added to micro tube. The mixtures were moved to cold cuvette and electroporated. After electroporation, the cell was added 1 mL of YPD media, moved to sterile micro tube then incubated at 30°C for 1 hour. The cell was centrifuged, and supernatant was discarded. The cell was re-suspended in 100 µL of sorbitol. It was inoculated in selective media, and incubated again at 30ºC for 15-16 hours.

### Expression of ads in Saccharomyces cerevisiae 

*Saccharomyces cerevisiae* cells harboring pBEVY-GU_*ads* were cultivated in SD-Ura+2% glucose at 30^o^C and 150 rpm. A 5 mL pre-culture was grown overnight. As much as 1% inoculum was seeded in 10 mL Erlenmeyer flask until OD_600_ reached 0.7. Thus the cells were harvested and washed twice with sterile water and re-suspended in SD-Ura+ 2% galactose for 24 h of induction time. Finally, the cells were harvested by centrifugation at 3000 rpm for 10 min. For protein analysis using SDS PAGE, the sample (equal amount of cell OD_600_ 2 per mL) was prepared using whole-cell lysis according to the protocol from Ida van der Klei lab with a necessary alteration.^21^ The cells were spun-down at 13000 rpm for 30 seconds, re-suspended in 300 μL of water and added 100 uL of 50% TCA. The samples were frozen at -80°C for a minimum of 30 min and spun for 5 min at 13000 rpm. They were washed twice with cold acetone and spin for 5 min at 13000 rpm. Acetone was evaporated and the pellet was air-dried. Thus, the pellet was re-suspended in 100 μL of 1% SDS and 0.1 N NaOH. Finally, 100 μL of 2x SDS-sample buffer was added and boiled for 5 min. For western blot, samples were run in 12% SDS PAGE gel. The gel was transferred to the PVDF membrane using eBlot protein transfer system (GenScript). The membrane was blocked using a blocking solution (TBST +5% skimmed milk) for overnight at 4°C, washed three times using TBST, followed by incubation with first 6x-His Tag Monoclonal antibody (4A12E4) for 1 h (1:1000 dilution, Thermo Fischer Scientific). Before incubation with the secondary antibody, the membrane was washed three times with TBST and incubated with HRP-conjugated Gt anti-ms IgG (H+L) secondary antibody for 1h (Invitrogen). The membrane was developed by the addition of ChromoSensor One Solution TMB Substrate GenScript.

## Results and Discussion

**Figure 1:**
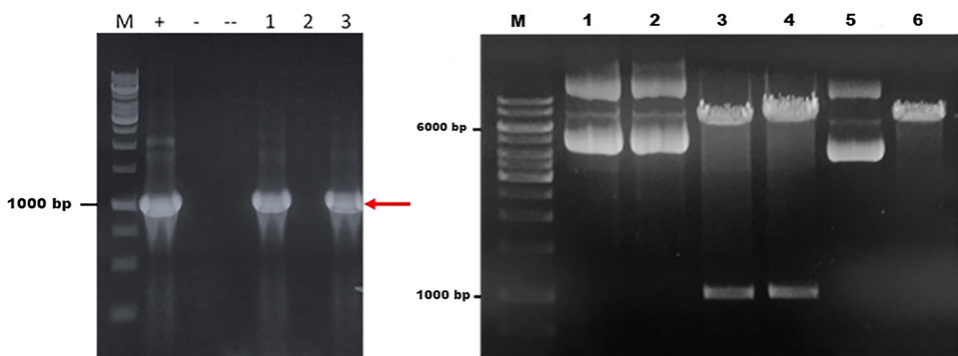
(a) Electrophoregram of PCR analysis of *Saccharomyces cerevisiae* M = DNA marker, (+) = pBEVY-GL_*fps* plasmid as a PCR template, (-) = pBEVY-GL plasmid as a PCR template, (--) = without PCR template, 1, 2 and 3 = colony number 1, 2 and 3. (b) Restriction analysis of pBEVY-GL_*fps*. 1,2 = pBEVY-GL_*fps* colony nr 1, 2; 3 = pBEVY-GL_*fps* /KpnI of colony 1; 4 = pBEVY-GL_*fps* /KpnI of colony nr 3; 5 = pBEVY-GL, and 6 = pBEVY-GL/KpnI.

The codon optimized fps gene was used in the study, since the original sequence was taken from *A. annua*. In order to be expressed in optimal condition, the DNA sequences should be optimized according to type of the host. The PCR of *fps* gene produced a single band at ~1000 bp showing the correct size for *fps* gene. Cloning of *fps* gene in PGEM T easy was successfully performed. This was done to confirm the quality of *fps* gene which was further used for transformation in *S. cerevisiae* through pBEVY vector. PCR analysis using three selected colonies showed that *fps* gene was successfully transformed in to *E. coli*. A clear band was shown from two colonies with the size of 1000 bp as the size of fps gene. The band was also shown by a positive control of pBEVY-GL_*fps*, while no band was shown by empty plasmid (Figure 1a). This means two out of three selected colonies contain pBEVY-GL_*fps* plasmid. The result was supported by restriction analysis using KpnI for colony 1 and 3 compare to empty plasmid pBEVY-GL. In Figure 1b , there is a different migration of pBEVY-GL_*fps* circular in lane 1 and 2 compared to empty pBEVY-GL plasmid in lane 5. This showed that there is a different on size between plasmid with and without insert gene.

According to virtual restriction analysis with Kpn1, colony nr 1 and 3 provide two bands in a size of around 6000-7000 bp and 1000-1500 bp, while pBEVY-GL plasmid without insertion provides only a band in a size of around 6000-7000 bp. The size of pBEVY-GL_*fps* is 7669 bp provided two bands after cut with Kpn1 with the size of 1357 bp and 6312 bp, while pBEVY-GL without insert only provides a band with the size of 6649 bp, which is a linear plasmid. It could be strongly indicated that the plasmid contained *fps* gene. Sequencing analysis showed that the gene was confirmed as *fps* gene.

**Figure 2:**
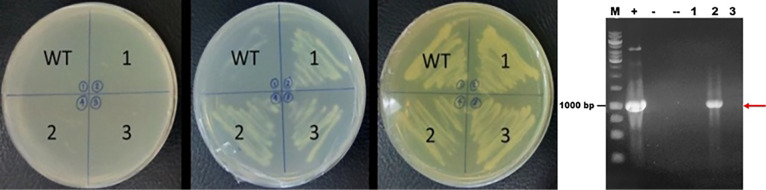
(a) The results of colonies selection in *Saccharomyces cerevisiae* wild type and transformant, WT = wild type, 1, 2, 3 = transformant colonies 1, 2 and 3 in the SD media without uracil (left), without leucine (middle) and YPD media (right). (b). PCR Electrophoregram of colonies M = DNA marker, (+) = pBEVY-GL_*fps* plasmid as a template (-) = *S. cerevisiae* wild type, (--) = without template

pBEVY-GL_*fps* was successfully transformed into *S. cerevisiae* using electroporation method and can be selectively screened using the auxotrophic selection system. In this research, the selection was done using SD media without leucine. Leucine is an essential amino acid required for growth. Since the pBEVY-GL plasmid has leucine marker, only transformants of *S. cerevisiae* harboring the plasmid are able to produce leucine and growth in that medium lacking leucine. The confirmation of selection was checked by the replication of colony in agar media without uracil, without leucine and on YPD media (Figure 2). Colonies growing in enriched media were compared to colonies growing in selective media. In the Figure 2a, the colonies could not grow in SD agar media without uracil, since the *S. cerevisiae* strain used could not produce uracil naturally. In SD agar media without leucine, the transformant colonies could only grow since it contains leucine gene pBEVY-GL_*fps* plasmid. In the YPD agar media, all colonies could grow well due to the amino acid rich-media. This showed that transformant colonies 1, 2 and 3 could contain empty pBEVY-GL or pBEVY-GL_*fps*. To confirm the existing of pBEVY-GL_*fps* plasmid, all colonies were used as templates for yeast PCR colonies. PCR analysis of transformant colonies showed that only colony 2 provide the band which is a correct size of *fps* gene (Figure 2b). Therefore, we conclude that the *S. cerevisiae* colony 2 contained *fps* gene. However, colony 1 and 3 did not provide any band, probably due to technical problem such as unsuccessful lysis of *S. cerevisiae* before used as PCR template.

**Figure 3:**
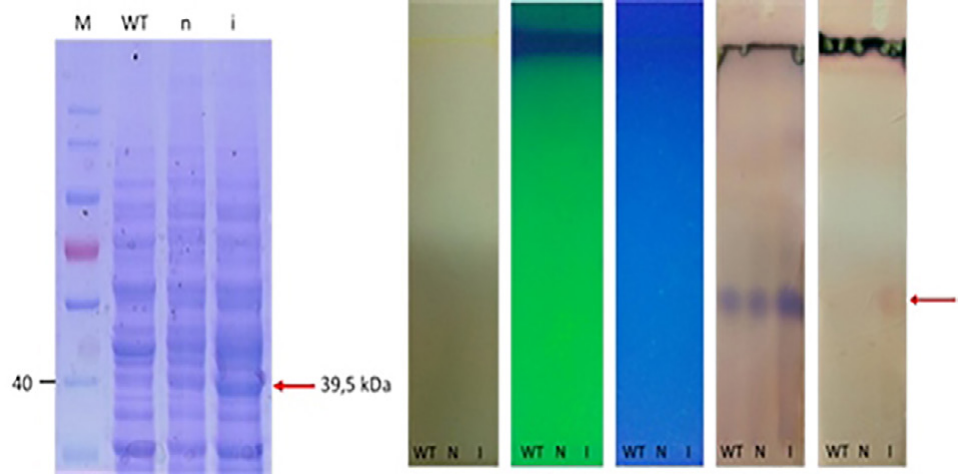
SDS PAGE electrophoregram of *fps* expression in *S. cerevisiae*. (left), TLC chromatogram of metabolites of recombinant *S. cerevisiae* (right) line M = Protein marker, WT = wild type, n = uninduced, I = induced

The production of fps enzyme as the results of the expression of *fps* gene in *S. cerevisiae* harboring pBEVY-GL_*fps* plasmid was done with induction of galactose. In the system of gene expression under galactose promotor; the presence of glucose could inhibit the galactose promotor, therefore raffinose was used instead of glucose as carbon source. The non-induced condition 2% raffinoses was used without the addition of galactose. Meanwhile, for induced condition, the 2% galactose was used as inducer as well as carbon sources. Induction was done during 18 hours (overnight) in order to induce the overexpression of *fps* enzymes. The enzyme expression was confirmed by SDS-PAGE electrophoresis. The results showed that there was a thick band with the size around 39.5 kDa from induced cell cultures compared to non-induced one and wild type (Figure 3). This indicated that the fps gene was successfully expressed in recombinant *S. cerevisiae* which was induced with 2% of galactose during 18 hours. This was supported with the preliminary check of metabolite by thin layer chromatography, and the induced cells produced higher level of metabolite than the others. This metabolite is supposed to farnesyl pyrophosphates as a result of converting IPP and DMAPP catalyzed by *fps* enzyme (Figure 3). However, the metabolite has to be further confirmed.

**Figure 4:**
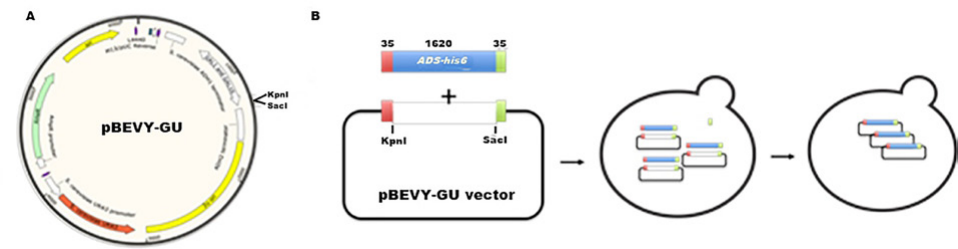
Homologous recombination-based cloning strategy of ads into *Saccharomyces cerevisiae*

Further experiment was continued to clone the next step gene on biosynthesis of artemisinin which was *ads* into *S. cerevisiae*. Similar to *fps* gene, the *ads* gene has also been optimized to be cloned in *S. cerevisiae*, since it is originally taken from *A. annua*. We used pBEVY GU which has uracil marker for selection and galactose promoter. pBEVY GU_*ads* was constructed using the homologous recombination-based cloning strategy (Figure 4). This was done after failing in cloning using classical cloning method as applied for *fps* gene above. This technique requires a homologous region between the inserted gene and its vector. To provide it, as many as 35 base pairs 5’ flanking region at both primers was added, corresponding to the homologous region of the pBEVY GU backbone at MCSII. Additionally, 6x-histidine tag was also added from the reverse plasmid for expression protein analysis.

**Figure 5:**
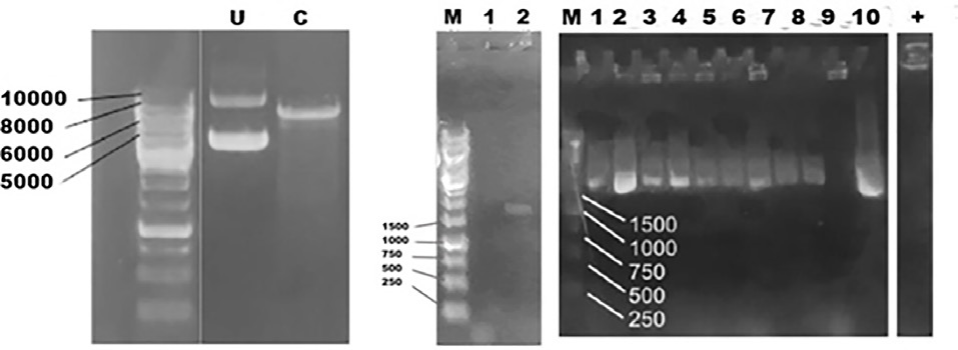
Electrophoregram of PCR of plasmid and PCR results U = uncut C = cut plasmid, M = DNA marker, 1 = negative control 2 = PCR result with ads band (a). PCR results of teen colonies of reacombinant *S. cerevisiae* harboring pBEVY-GU_ads (b)

To confirm the expected *ads* gene for preparation prior to cloning, the PCR analysis using template pUC57_ads and a pair of primers for ads showed the right size of *ads*. An agarose gel showed that the PCR produced only one specific band at the expected size ~1700 bp (Figure 5a). It showed that the *ads* gene was cut from the plasmid of pUC57_ads and ready for transformation into pBEVY vector. We observed no band at the negative control. Restriction analysis for cut and un-cut pBEVY plasmids also showed the expected size. The purified fragment DNA from ads and cut plasmid was then mixed (ratio of vector and insert = 1: 4 v/v) and successfully transformed the pre-treated *S. cerevisiae* by electroporation and plated at selective media SD-Ura + 2% glucose. The transformant colonies were re-plated to the new agar media for the second selection. For confirmation of yeast cloning, we did colony PCR of yeast transformants and showed that 9 out of 10 colonies showed positive result (Figure 5b) with expected size. For sequencing purpose, 2 positive colonies were selected. The total DNA of were isolated from these 2 colonies and transformed them into *E. coli* TOP10 F’. Fifteen of *E. coli* colonies were analyzed by colony PCR and 4 of them showed positive result (~26%). The low yield of colony PCR of *E. coli* transformant could be due to the heterogeneity of the yeast cell harboring recombinant plasmid.

Next, two plasmids were purified and checked with restriction analysis before being sent to sequencing. XbaI was used to cut an empty and the recombinant one. The pBEVY GU that only has one site of XbaI will give a 6145 bp whilst the correct recombinant plasmid will produce two bands at 6149 and 1497 bp. This showed that the ads was successfully cloned in *S. cerevisiae*. Further check with sequencing analysis supported the conformation.

**Figure 6:**
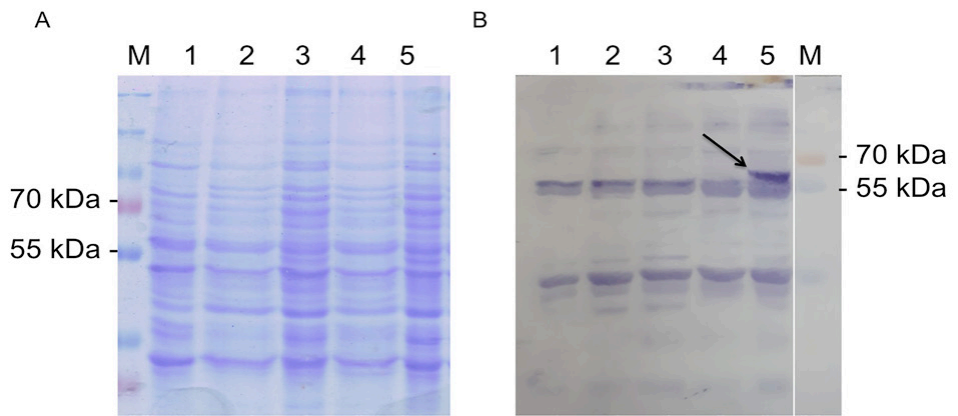
SDS PAGE electrophoregram (left) and western blot (right) of *ads* expression in *S. cerevisiae*. M = Protein marker, 1 = wild type, 2 and 3 = uninduced empty vector, 4 = uninduced pBEVY_GU harbouring *ads*, 4 = induced, I = induced pBEVY_GU harbouring ads

Expression of *ads* was induced by addition of galactose. Here, we used 2% galactose not only as the inducer but also as carbon source. As negative control, we also expressed the wild type, and cells harboring empty plasmid. Unfortunately, the expression of *ads* analyzed with SDS PAGE solely is not distinguishable, then we used western blot against anti his antibody in the crude extract of yeast cell and finally the ads enzyme could be detected at its expected molecular weight (65 kDa) (Figure 6). The low concentration of protein was probably one of the reasons why the protein could not be detected by SDS PAGE. However, using western blot with anti his is more specific to the target ads gene. Thus we conclude that ads gene was successfully cloned and expressed in *S. cerevisiae*. We do observe multiple unspecific bands in the western blot including in the crude extract of wildtype cells. This has to be taken into account when performing western blot using his tag antibody, especially when the protein of interest has the same molecular weight of the protein background. Finally, we conclude that the ads protein is successfully cloned and expressed in *S. cerevisiae* at the expected size of ~65 kDa.

The direct cloning method in yeast is simpler, cost-efficient and less time consuming. We are taking advantage of the ability of yeast cell to do recombination event as genome repair system by introducing overlapping region between the insert gene and the vector backbone. This homologous recombination mechanism facilitates mainly by Rad52 repair group.^22^ Thus, the strain background used to perform yeast cloning should not contain any essential mutation in the Rad52 repair group. For broader usage of this technique, other researcher has made a yeast cassette that can construct any type of plasmid and any gene by introducing an original replication in yeast and selective marker.^23^

## Conclusion

Two key enzymes encoded genes of antimalarial biosynthesis which are farnesyl phospate synthase (*fps*) and amorpha-4,11-diene synthase (*ads*), have been successfully transformed into *Saccharomyces cerevisiae*. These two enzymes could be expressed in recombinant *S. cerevisiae* harboring pBEVY-GL-*fps* and pBEVY-GU_*ads* plasmids. SDS PAGE electropherogram showed that *fps* enzyme could be visualized with coomassie blue at the correct size of 39.5 kDA, while the *sds* enzyme could not be visualized with coomassie blue, but with a blotting using anti his antibody was successfully visualized with the correct size of 6 kDa

## Ethical Issues

Not applicable.

## Conflict of Interest

The authors confirm that this article content has no conflicts of interest.

## Acknowledgments

The author would like to thanks the Directorate of Research and Community services, Ministry of Research, Technology and Higher Education, Republic of Indonesia and Bandung Institute of Technology for financial support of the research. We also would like to thank Dr. Dessy Natalia’s Lab for their kindness in sharing and supporting the blot transfers equipment and chemical for western blot.
